# Artificial Intelligence-Driven Algorithm for Drug Effect Prediction on Atrial Fibrillation: An *in silico* Population of Models Approach

**DOI:** 10.3389/fphys.2021.768468

**Published:** 2021-12-06

**Authors:** Ana Maria Sanchez de la Nava, Ángel Arenal, Francisco Fernández-Avilés, Felipe Atienza

**Affiliations:** ^1^Department of Cardiology, Hospital General Universitario Gregorio Marañón, Instituto de Investigación Sanitaria Gregorio Marañón (IISGM), Madrid, Spain; ^2^Centro de Investigación Biomédica en Red de Enfermedades Cardiovasculares (CIBERCV), Madrid, Spain; ^3^ITACA Institute, Universitat Politécnica de València, València, Spain; ^4^Facultad de Medicina, Universidad Complutense de Madrid, Madrid, Spain

**Keywords:** atrial fibrillation, computational models, decision tree, drug safety, safety pharmacology

## Abstract

**Background:** Antiarrhythmic drugs are the first-line treatment for atrial fibrillation (AF), but their effect is highly dependent on the characteristics of the patient. Moreover, anatomical variability, and specifically atrial size, have also a strong influence on AF recurrence.

**Objective:** We performed a proof-of-concept study using artificial intelligence (AI) that enabled us to identify proarrhythmic profiles based on pattern identification from *in silico* simulations.

**Methods:** A population of models consisting of 127 electrophysiological profiles with a variation of nine electrophysiological variables (G_*Na*_, I_*NaK*_, G_*K*1_, G_*CaL*_, G_*Kur*_, I_*KCa*_, [Na]_*ext*_, and [K]_*ext*_ and diffusion) was simulated using the Koivumaki atrial model on square planes corresponding to a normal (16 cm^2^) and dilated (22.5 cm^2^) atrium. The simple pore channel equation was used for drug implementation including three drugs (isoproterenol, flecainide, and verapamil). We analyzed the effect of every ionic channel combination to evaluate arrhythmia induction. A Random Forest algorithm was trained using the population of models and AF inducibility as input and output, respectively. The algorithm was trained with 80% of the data (*N* = 832) and 20% of the data was used for testing with a *k*-fold cross-validation (*k* = 5).

**Results:** We found two electrophysiological patterns derived from the AI algorithm that was associated with proarrhythmic behavior in most of the profiles, where G_*K*1_ was identified as the most important current for classifying the proarrhythmicity of a given profile. Additionally, we found different effects of the drugs depending on the electrophysiological profile and a higher tendency of the dilated tissue to fibrillate (Small tissue: 80 profiles vs Dilated tissue: 87 profiles).

**Conclusion:** Artificial intelligence algorithms appear as a novel tool for electrophysiological pattern identification and analysis of the effect of antiarrhythmic drugs on a heterogeneous population of patients with AF.

## Introduction

The first-line treatment for atrial fibrillation (AF) is antiarrhythmic drugs, although undesirable proarrhythmic effects have been identified in some cases. The response to these drugs is highly dependent on the specific baseline electrophysiological characteristics of the patient. In this framework, safety pharmacology has emerged as a new field in cardiac arrhythmias with the aim of identifying the drug hazard (Kraushaar et al., 2012; Mirams et al., 2012; Davies et al., 2019) by detecting the probability of triggering an arrhythmia. Different tests have been designed with the objective of determining the proarrhythmicity of a given compound (Falk, 1989; Crumb et al., 2016; Passini et al., 2017).

Consequently, variability is an important factor to be studied and analyzed to understand its dependency between the specific characteristics of the patient and the effect of the drug. In this scenario, several studies have included and incorporated variability in mathematical approaches by means of a population of models (Britton et al., 2013; Liberos et al., 2016; Muszkiewicz et al., 2016; Bai et al., 2021) to account for the electrophysiological heterogeneity presented in a real population of patients. Other approaches have also been implemented in more recent studies such as the CiPA initiative, which combines *in vitro*, *in silico* and clinical data to build a platform that can be used for testing new drugs and their potential harmful effects (Sager, 2014) in ventricular myocyte models. In addition, studies have also explored electrophysiological variability to identify potential currents involved in AF triggering and maintenance (Ellinwood et al., 2017; Bai et al., 2020). However, these approaches usually focus at the unicellular level or present low variability at an electrophysiological level in the field of AF.

Anatomical complexity has also been incorporated including *in silico* studies using two-dimensional (2D) and three-dimensional (3D) structures rather than unicellular approaches to evaluate the proarrhythmicity of anatomical structures (Varela et al., 2016). Within this framework, several studies have identified specific currents or biomarkers that can explain or characterize the proarrhythmicity of a compound. New scenarios considered at this stage the use of sophisticated statistical methods that can, not only identify isolated biomarkers but groups or clusters that better react to a specific treatment. Although the use of a population of models for the evaluation of proarrhythmicity usually presents a broad representation of the electrophysiological characteristics of these patients, other variables that highly influence the arrhythmia induction and maintenance should be explored. For example, other factors aside from ionic remodeling that can affect AF maintenance can be found in the literature such as the size of the atria, which has been previously identified as an increased probability of triggering an arrhythmia for bigger or dilated tissues. With all these considerations, a new dimension is included in the simulations, introducing anatomical variability into the important factors underlying arrhythmia maintenance (Nattel et al., 2008; Qureshi et al., 2014).

However, and despite the complexity included in all these studies, the identification of new biomarkers or patterns is still challenging and present low accuracy metrics. Our hypothesis was that artificial intelligence (AI) could extract patterns or clusters from *in silico* simulations that can help to better predict the effect and risk of antiarrhythmic therapies.

In this study, a population of models with AF, including electrophysiological and anatomical variability, was used to study the effect of different drugs on the arrhythmia behavior and was then analyzed by means of AI algorithms. Our research was built on previous studies using a population of models (Liberos et al., 2016) showing the importance of specific currents on the drug effect. In addition, previous studies have identified one ionic current or a combination of them (Pandit et al., 2005; Dobrev et al., 2011; Jiang et al., 2017) but none of them have implemented an algorithm that specifies the threshold for each variable of the ionic profile. Our algorithm was developed as a proof of concept of AI applied to the population of models guiding the identification of the effect of drug therapy on a heterogeneous population.

## Materials and Methods

### Electrophysiological Variation: Description of the Population of Models

To obtain data for the population of models, samples from the right atrial appendages from 149 patients diagnosed with chronic AF in which antiarrhythmic medication was interrupted before the study were available (Wettwer et al., 2013; Sánchez et al., 2014).

Briefly, patch-clamp was performed in all the samples obtaining the values for different currents. A total of six biomarkers were used to quantify variability in action potentials (AP) including AP duration at 20, 50, and 90% of repolarization (APD20, APD50, and APD90, respectively), AP amplitude (APA), resting membrane potential (RMP), and AP plateau potential at 20% of APD20 (V20). The maximum and minimum values of these biomarkers at a pacing frequency of 1 Hz are presented in Supplementary Table 1.

To build the computational population of human AF models, different combinations of ionic currents were generated from the experimental data described earlier. In further detail, Latin Hypercubic Sampling (LHS) (McKay et al., 1979) and the baseline AF model developed by Koivumaki et al. were used for its generation.

A total of nine parameters were varied from –50 to +100% of their original value: fast sodium (Na^+^) ionic conductance (g_*Na*_), sodium-potassium ion (Na^+^–K^+^) pump (I_*NaK*_), inward rectified K^+^ current (gK1), L-type calcium ionic conductance (g_*CaL*_), ultrarapid outward ionic conductance (g_*Kur*_), Ca^2+^-dependent K^+^ current (I_*KCa*_), and Na^+^ and K^+^ extracellular concentration and the diffusion coefficient of the reaction-diffusion equation (Supplementary Figure 2). LHS produced 500 different combinations of these nine parameters from the initial 149 patients. Simulations for these 500 profiles were calculated on planes of 8 × 256 nodes to evaluate the AP metrics. The models were simulated by pacing at 1 Hz (using a 3 ms stimulus duration, twice diastolic threshold amplitude). The APs of three cells along the plane (cells 500, 620, and 748) were analyzed following a train of 15 periodic stimuli, and the last 5 periodic stimuli were considered in order to ensure a steady-state. Only the models that fitted into the experimental constraints (Supplementary Table 1) were considered representative human electrophysiological models for the final population, resulting in 127 final profiles (Simon et al., 2017).

### Chronic Atrial Fibrillation Electrophysiological Cellular Model

Different atrial models have been described at a unicellular level to characterize the electrophysiological response. In this study, we implemented the Koivumaki model with Skibsbye modifications (Skibsbye et al., 2016) that included a reformulation in sodium current to characterize sodium channel inactivation, adjusted transient outward potassium current and L-type Ca^+^ current, and included the small conductance calcium-activated potassium current (I_*KCA*_). In addition, the model used for simulations included AF remodeling, achieved by modifying the following ionic currents: L-type Ca^2+^ (I_*CaL*_) decreased by 55%, transient outward current (Ito) decreased by 62%, rapid delayed rectifier potassium channel (I_*Kur*_) decreased by 38%, inward-rectifier potassium channel (I_*K*1_) increased by 62%, Na/Ca exchange current (I_*NCX*_) increased by 50%, expression of sarcoplasmic/endoplasmic reticulum Ca^2+^-ATPase (SERCA) decreased by 16%, phospholamban to SERCA increased by 18% and sarcopilin to SERCA decreased by 40% as described in Koivumaki et al. (2014). This model was used for both the calibration of the population of models previously described and the rest of the experiments in the study.

### Anatomical Characterization: Monodomain Equation and Tissue Size

Simulations were performed on 2D planes mimicking a sheet of cardiac tissue. Two different tissue sizes were implemented for this study: one square plane corresponding to a normal atrium (16 cm^2^, 400 × 400 nodes) and another square plane corresponding to a dilated atrium (20.25 cm^2^, 450 × 450 nodes) (Kou et al., 2014).

**FIGURE 1:**
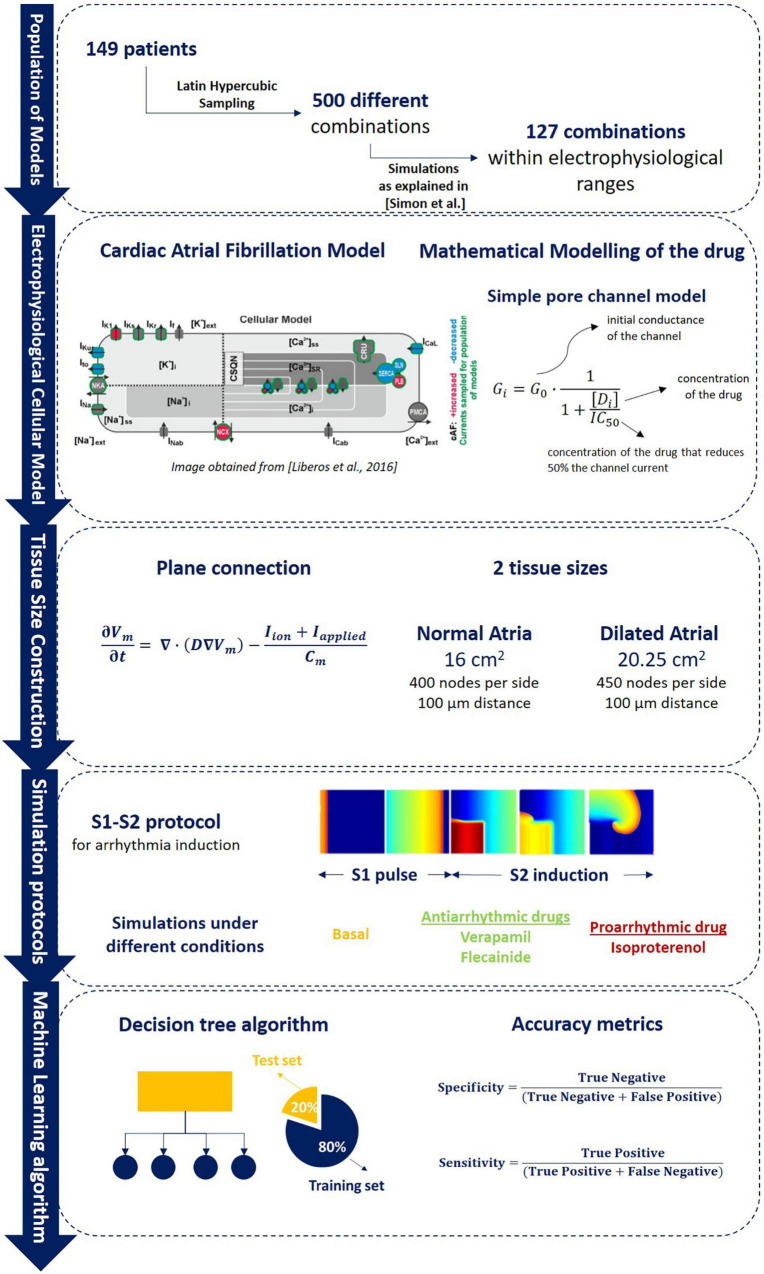
Overall methodology description including (1) population of models calibration as described in Simon et al. (2017) in which samples from 149 patients were obtained to evaluate 500 different ionic combinations that resulted in a final pool of 127 electrophysiological profiles that composed the population of models (section “Electrophysiological Variation: Description of the Population of Models”); (2) brief description of the Koivumaki electrophysiological model used and the mathematical modeling of the drug as described (section “ Chronic Atrial Fibrillation Electrophysiological Cellular Model”); (3) connection between different cells for plane simulations and tissue size used for each of the 2D planes used (sections “Anatomical Characterization: Monodomain Equation and Tissue Size” and “Mathematical Modeling of the Drug”); (4) S1–S2 arrhythmia induction protocol simulation and pharmacological compounds simulated during the experiments (section “Simulation Protocols”); (5) Artificial intelligence algorithm training (80% of data) and testing division (20% of data) for the identification of proarrhythmic profiles (section “Random Forest Algorithm for Atrial Fibrillation Maintenance Prediction”).

To connect the cells within the plane, the monodomain reaction-diffusion equation was implemented, assuming that tissue behaves as a functional syncytium where membrane voltage is propagated smoothly (Clayton and Panfilov, 2008):


$$\frac{\partial V_{m}}{\partial t}=\nabla \cdot \left(D\nabla V_{m}\right)-\frac{I_{\mathrm{ion}}+I_{\mathrm{applied}}}{C_{m}}$$

where the ∇ corresponds to the gradient operator and *D* a diffusion coefficient with units distance^2^ time^–1^. By using this monodomain simplification, the tissue is considered to have an unlimited extracellular medium, so the extracellular resistivity can be neglected. The extracellular medium is isopotential and equal to zero for simplicity. Consequently, the membrane potential is the same as the intracellular potential. Planes were fully connected as shown in Figure 1, not including structures such as the pulmonary veins. The value of the diffusion constant, referred to as D in the above equation, was varied among the different profiles as part of the variability included in the population of models.

### Mathematical Modeling of the Drug

Both antiarrhythmic and proarrhythmic drugs were evaluated in the electrophysiological population of models in order to characterize the effect according to tissue size and ionic currents. Three different drugs were studied presenting different mechanisms and effects, namely, verapamil, flecainide, and isoproterenol. Briefly, verapamil is an antiarrhythmic drug that acts as a calcium blocker, flecainide is an antiarrhythmic drug that acts as a sodium blocker and isoproterenol is a proarrhythmic agent (β receptor agonist) that increases intracellular calcium. All three drugs are currently used in clinical practice and their effect, although established as proarrhythmic or antiarrhythmic, can vary from patient to patient (Bassett et al., 1997). A simple pore channel equation was used for drug implementation at the computational level including three channels for each drug that was modeled according to experimental IC50 values to calculate the block of the channel (Dempsey et al., 2014). The model used is described as follows:


$$G_{i}=G_{0}\times \frac{1}{1+\frac{\left[D_{i}\right]}{IC_{50}}}$$

where the G_0_ represents the initial conductance of the channel for each of the profiles in the population of modes, [*D*_*i*_] corresponds to the concentration of the drug and IC_50_ is the concentration of the drug that reduces by 50% the channel current. For verapamil and flecainide, values from Crumb et al. (2016) were implemented. Isoproterenol was modeled to increase the permeability of the calcium current as stated in Vescovo et al. (1989). The numerical values for each modeled drug can be observed in Table 2, including the concentration at which the drug was modeled.

**TABLE 1:** Profiles maintaining rotational activity for different drug and tissue sizes.

		Atrial fibrillation	Sinus rhythm
Small	Basal	80 (63.00%)	47 (37.00%)
	Flecainide	37 (29.13%)	90 (70.87%)
	Verapamil	37 (29.13%)	90 (70.87%)
	Isoproterenol	88 (69.29%)	39 (30.71%)
Dilated	Basal	87 (68.50%)	40 (31.50%)
	Flecainide	63 (49.61%)	64 (50.39%)
	Verapamil	64 (50.39%)	63 (49.61%)
	Isoproterenol	94 (74.02%)	33 (25.98%)

*For each tissue size and basal/drug condition, the table specifies the number of profiles with AF inducibility. The percentage of the profiles with reentry is specified in parenthesis for every 127 profiles simulated in each case.*

**TABLE 2:** Parameters for modeling the drug effect include drug concentration, IC50 for the three specific channels modeled.

	Drug concentration (mM)	Nav1.5-peak	hERG	Cav1.2
Flecainide	2.0 e-04	6.7	0.7	20
Verapamil	5.0 e-04	1.0	0.7	0.1
Isoproterenol	8.0 e-02	–	–	20*

*Corresponds to EC50.

### Simulation Protocols

Simulations were performed implementing differential equations computed with a time step of 1 microsecond for the Euler method using in-house software written in C++ with CUDA parallelization and solved with a NVIDIA TESLA C2057 GPU, NVIDIA Corporation, Santa Clara, CA, United States. Rush Larsen method was developed for cell models as it offers stability to the problem by calculating the exact solution for the gating variables. Since all the equations governing the gating variables have a similar structure, the method uses the following expression to solve them:


$$w_{i}^{j+1}=e^{ai\left(v\right)h}\left(w_{i}^{j}+\frac{b_{i}\left(V\right)}{a\left(V\right)}\right)-\frac{b_{i}\left(V\right)}{a\left(V\right)}$$

Consequently, all equations for gating variables were solved by the previous expression whereas the rest of the equations were solved by the forward Euler Method.

Planes were simulated for a total of three impulses (S1) at 1 Hz followed by a fourth one (S2) for arrhythmia initiation. S2 was induced in the cells of the inferior left section of the plane, producing reentry in part of the models. For a given combination of ionic channels in the population of models, arrhythmia was induced when >1 complete rotational activity followed the S1–S2 induction protocol. In addition, rotor tracking was studied for two specific profiles that will be discussed in the “Result” section. The methodology for rotor tracking has already been described in previous publications of the group (Rodrigo et al., 2014). Briefly, phase maps of the simulations were calculated by using the Hilbert transform from which singularity points were calculated. A singularity point (SP) was defined as the point in a phase map that is surrounded by phases from 0 to 2π. Only those singularity points that were present for the duration of at least 1 full rotation were considered, as described in Rodrigo et al. (2014). Rotor track was defined as the connection between SPs across spherical layers at a given time. Only filaments that completed at least 1 rotation on the outermost surface were considered.

### Random Forest Algorithm for Atrial Fibrillation Maintenance Prediction

A total of 1,016 simulations were computed in this study corresponding to all different combinations of the population of models (127 profiles) simulated in different tissue sizes (two tissue sizes) and four different conditions (baseline conditions, two antiarrhythmic drugs, and one proarrhythmic drug), creating a database with different ionic conductance combinations and tissue size.

Random Forest, which is a decision algorithm consisting of a multitude of decision trees at training time, was implemented to output two possible outcomes: induced or non-inducible AF. This algorithm was trained including the eight variables of the population of models as an input and the presence of AF form simulations as an output to evaluate patterns that may lead to AF maintenance. The algorithm was trained with 80% of the data (*N* = 832) and 20% of the data was used for testing with a 5k-fold cross-validation.

## Results

### Atrial Fibrillation Induction on the Population of Models

From the complete population consisting of 127 different electrophysiological profiles, 80 profiles maintained the reentrant activity at baseline conditions in the normal tissue size and 87 in the dilated atrium. Complete quantification of the profiles maintaining reentry can be observed in Table 1, including the effect of the three simulated drugs. As shown, dilated tissue increased the number of profiles maintaining reentry in all cases, independently of the presence and type of drug.

**FIGURE 2:**
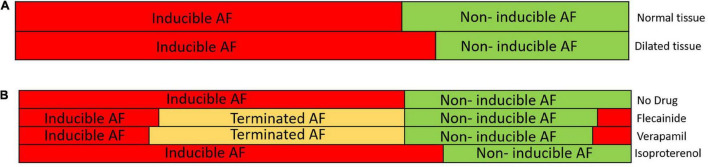
**(A)** Distribution of the population of models with induced and non-inducible atrial fibrillation (AF) for the normal tissue (*top*) and the dilated tissue (*bottom*). The red color shows the proportion of the models with inducible AF during simulation and the green color shows the proportion of the models with non-inducible AF. **(B)** Distribution of the population of models for the normal size tissue under the 4 studied conditions (no drug, flecainide, verapamil, and isoproterenol) with the proportion of inducible AF (red), non-inducible AF (green) in each of the cases. Terminated AF (yellow) corresponds to the profiles that, due to the electrophysiological changes induced by the drug, the reentry was not inducible.

**FIGURE 3:**
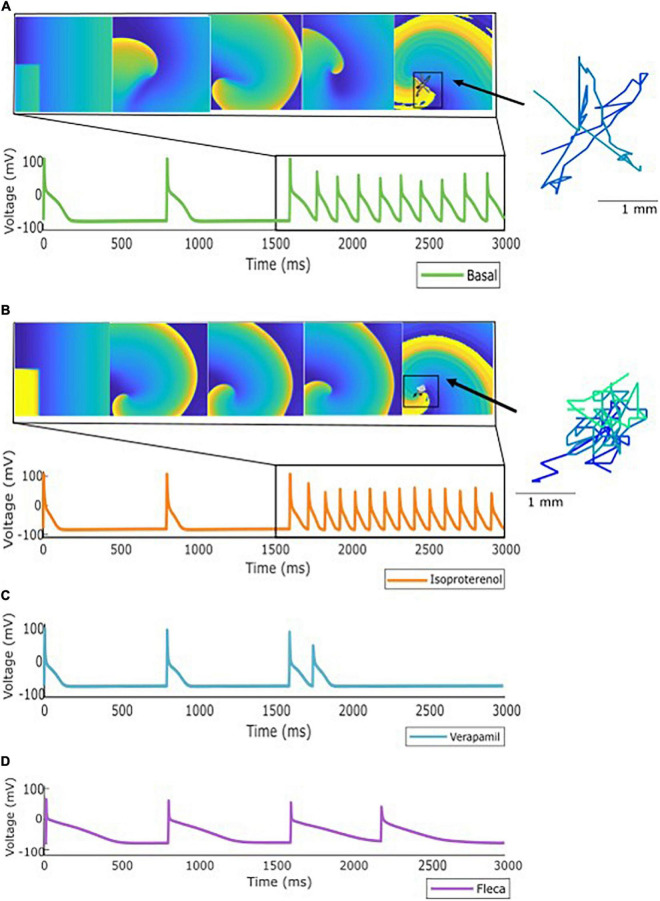
Specific profile from the population of models showing: **(A)** Inducible AF after stimulation protocol. **(B)** Inducible AF due to effect of isoproterenol, showing an increase on the activation dynamics. **(C)** Antiarrhythmic effect of verapamil and **(D)** Antiarrhythmic effect of flecainide. As an example, rotor meandering is shown in panels **(A,B)**, exemplifying the effect of isoproterenol in arrhythmia stabilization.

**FIGURE 4:**
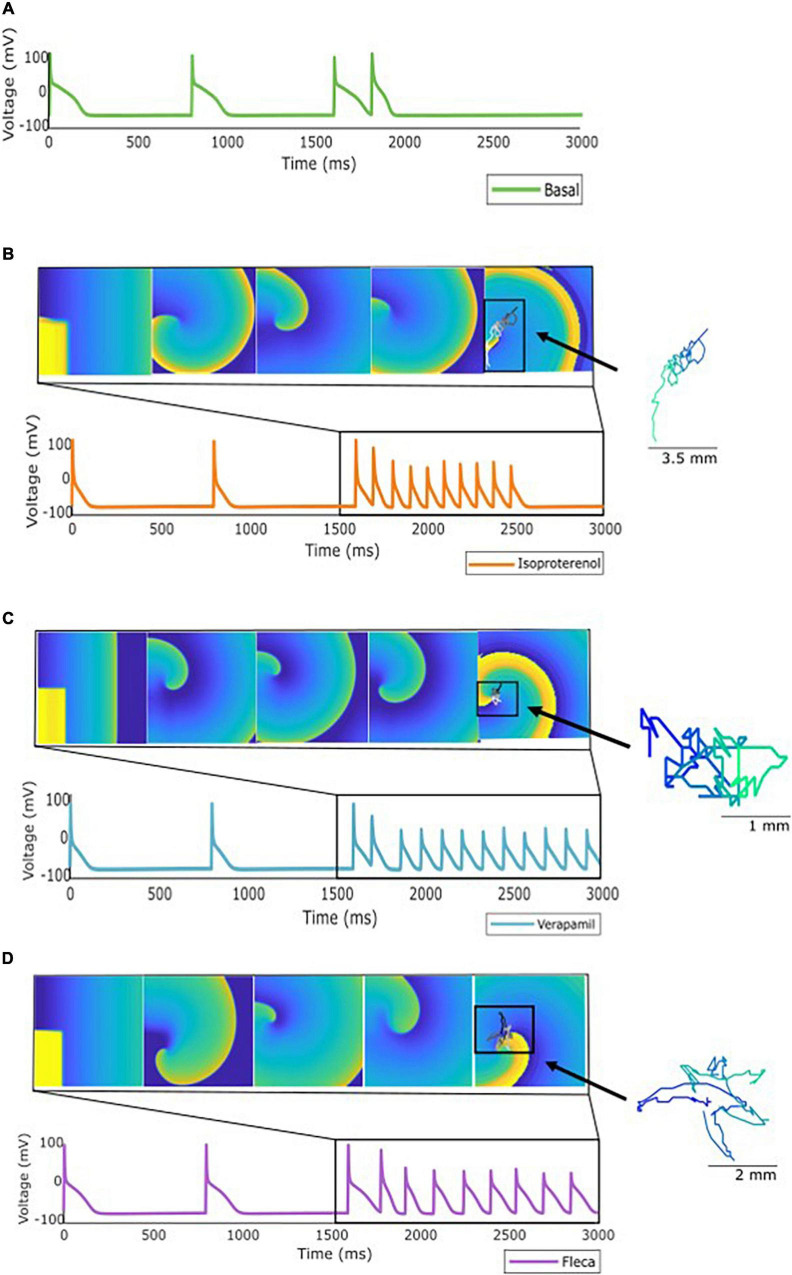
Specific profile from the population of models showing: **(A)** No AF induction using the stimulation protocol described. **(B)** Induction but not maintenance of AF when the simulation was repeated under the effect of isoproterenol. **(C)** Induction and maintenance of AF under the effect of verapamil, and **(D)** induction and maintenance under the effect of flecainide. As an example, rotor meandering is shown in panels **(B–D)** exemplifying the effect of the drugs in arrhythmia stabilization.

The analysis of the simulations resulted in the identification of profiles that responded differently to the arrhythmia induction under the effect of the drug, as can be observed in arrhythmia induction Figure 2. Non-inducibility of the arrhythmia was observed in the majority of the profiles when the antiarrhythmic compounds, verapamil, and flecainide were used. However, in some of the profiles that did not induce the arrhythmia at baseline, the addition of one of those drugs induced rotational activity (Figure 2B). Specifically, the addition of flecainide gave rise to non-inducibility of the arrhythmia in 51 profiles and induced AF in seven profiles that were non-inducible at baseline. For verapamil, 53 and 8 profiles were non-inducible and induced the arrhythmia, respectively, in normal tissue size samples. Interestingly, all the profiles that showed proarrhythmic and antiarrhythmic effects in the flecainide scenario presented the same behavior in the verapamil scenario. Although verapamil and flecainide showed similar results, the overall action potential morphology was significantly different for the same profile under the effect of these drugs, as it can be observed in Figures 3, 4, where the curvature of the action potential is modified due to the effect of the drug at the ionic level. Isoproterenol showed a proarrhythmic effect increasing the number of AF maintaining profiles in eight cases.

### Antagonistic Effects Were Observed for the Same Drug Among the Population of Models

Antagonistic effects of both antiarrhythmic and proarrhythmic drugs were observed on the population of models. Figure 3 presents a specific ionic profile simulated for all four conditions (basal and three drugs) in which reentry was maintained over time in the basal scenario. When the simulation was repeated under the effect of verapamil or flecainide, the arrhythmia was not induced. With the addition of isoproterenol, the arrhythmia was, not only induced but rotational activity presented a higher activation frequency.

For another specific profile shown in Figure 4, antagonistic effects were observed as follows: for a profile in which arrhythmia was not induced under basal conditions, verapamil and flecainide showed a proarrhythmic effect, meaning that the arrhythmia was induced, while isoproterenol did not induce the reentry for the complete simulation. Furthermore, for these aforementioned profiles, rotor tracking shows the lower area and higher complexity of the simulations in which the arrhythmia was maintained over time, exhibiting the stability of the reentry. Flecainide and verapamil terminated AF for the majority of the profiles in which AF was maintained under basal conditions, whereas isoproterenol has induced AF in new profiles which were not inducible in basal conditions. Interestingly, a small proportion of models presented AF induction under the effect of verapamil and flecainide (Figure 2B) despite the absence of AF induction at baseline.

### Machine Learning Algorithms Help Understand and Predict the Effect of the Ionic Channel

**FIGURE 5:**
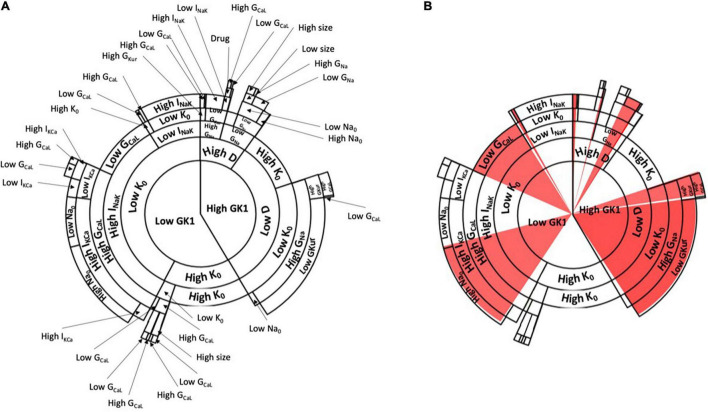
**(A)** Decision tree algorithm of the change in conductances based on the population of models with the information of the examples presented in Figures 1, 2. The decision tree starts from the center of the circle and continues in an outward trend including all the variables described in the population of models. Specific paths of the decision algorithm led to induced AF, which is shown in red (inducible AF) in panel **(B)**.

We generated a final database of 1,016 simulations that included the basal and drug administration state for both tissue sizes. Therefore, these data represented: (1) the variables of the population of models, (2) the electrophysiologic change conferred by the drug administration; (3) the changes between normal and dilated tissue size. Then, the database was processed in order to train and calibrate a decision algorithm for drug effect prediction. As a result, the Random Forest algorithm, shown in Figure 5, was obtained. The algorithm had in total seven different consecutive layers, shown in Figure 5A that cluster similar profiles together for prediction of AF induction based on the conductance values in the form of a sunburst diagram. In this diagram, each level is represented by a concentric circle containing one or more variables from the population of models, where each level presents threshold values for decision making. To evaluate a specific profile, the decision algorithm starts the clustering process from the most inner circle, that corresponds to the G_*K*1_ variable. For each variable, a threshold value has been defined that has to be compared with the value of the variable for the specific profile that is being evaluated. Specific values for each threshold can be consulted in the Supplementary Figure 1. For example, in the case of G_*K*1_, this value corresponded to 9.12%. If the value of the profile is higher than 9.12%, the next level to be considered will correspond to the right part of the diagram and the next variable (High G_*K*1_) to continue the characterization, corresponding to the diffusion variable. In contrast, if the value of the profile is lower than 9.12% for G_*K*1_, the next level to be considered will correspond to the left part of the diagram and the next variable, identified as K_0_. This process should be completed until the last layer or circle of the diagram, finding the path or cluster to which a given profile belongs. Figure 5B shows the paths or clusters that have been identified as inducible AF by the algorithm.

Interestingly, if the paths of clusters from the diagram are analyzed, it can be observed that one of the main contributions that lead to AF inducibility is the combination of profiles with high conductance of the inward rectifier K^+^ channel (K1), low diffusion, high concentration of extracellular potassium, high conductance for the sodium channel and low conductance of the ultra-rapidly activating delayed rectifier current (I_*Kur*_). Another combination that led to AF was low conductance value for I_*K*1_, low concentration of extracellular potassium, high conductance for the sodium-potassium pump, high conductance for the slow calcium channel, high conductance for the calcium-potassium pump, and high conductance for the I_*Na*_ channel.

**FIGURE 6:**
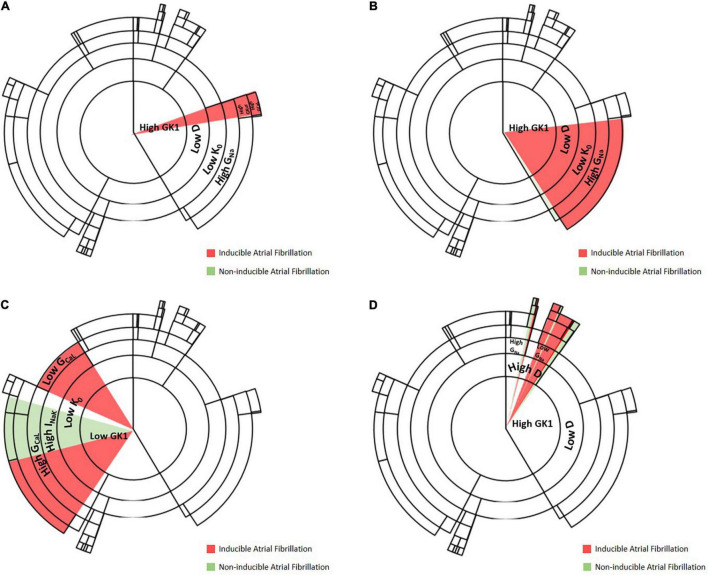
Examples of change in channel conductivity that lead to a change in induced/non-inducible AF. **(A)** Level at which increase of calcium channel conductance permeability results in maintenance of AF, an example of the proarrhythmicity of isoproterenol. **(B)** Level at which decrease of sodium conductance results in AF non-inducibility, an example of antiarrhythmic effect of flecainide. **(C)** Level at which increase or decrease of calcium channel permeability results in AF induction, an example of verapamil being antiarrhythmic and isoproterenol having a proarrhythmic effect. **(D)** Level at which increase of sodium permeability increases the probability of AF inducibility.

Figure 6 shows an example of how the change in conductance of specific channels can affect the final path or cluster to which a specific profile belongs, altering the final outcome of the decision tree algorithm. In this figure, specific pathways of the decision tree are exemplified in each of the panels, highlighting how the change due to the addition of the drug affects specific levels that, depending on the final permeability of the channel, may be fundamental for the induction and maintenance of the rotational activity. Panels A, C highlight changes in calcium channel conductance, that can be affected by the addition of verapamil or isoproterenol, as both drugs were modeled to respectively, decrease or increase the permeability of this channel. Specifically, Panel A exemplifies how the increase in the permeability of the calcium channel, by the addition of isoproterenol, has a proarrhythmic effect. Conversely, a profile that presents high conductance of the calcium channel can be reduced by adding verapamil and changing to a state of non-inducibility of AF. Panel C shows another example in which the decrease of calcium permeability results in AF inducibility and the increase of calcium permeability increases the probability of AF non-inducibility.

Examples for flecainide, which affects sodium channel permeability, are shown in Panels B, D. Specifically, Panel B shows an antiarrhythmic effect of the addition of flecainide, by blocking sodium channels. Panel D shows a level of the decision tree algorithm at which, by changing sodium channel permeability, the probability of AF non-inducibility increases. Thus, the probability of inducing AF will be higher for those profiles with lower sodium permeability, showing a proarrhythmic effect of the drug.

## Discussion

In this study, we present a new algorithm that identified and clustered the combination of channel conductivities that promoted arrhythmia initiation. The algorithm was calibrated with *in silico* data obtained from 2D simulations in two different size planes simulated on a population of models under the effect of three different drug effects (isoproterenol, verapamil, and flecainide) resulting in 1,016 different simulations. The main result of the study was the following: first, we proved, in a population of the simulation environment of the models, that dilated tissues are more prone to induce AF and that the effect of a given drug can differ from one profile to another depending on the specific expression of the currents. Finally, we also proved that all this information can be used to train a machine-learning algorithm to predict the AF inducibility of the tissue.

### Variability on Atrial Fibrillation Simulations: Ionic and Tissue Size Variation

The population of models has been widely used in the cardiac electrophysiology field for safety pharmacology, providing new platforms for the assessment of proarrhythmic effects of drugs (Passini et al., 2017; Kügler, 2020). In this study, we have analyzed how variations of the electrophysiological and anatomical characteristics can affect AF inducibility at the 2D level. Electrophysiological variability was introduced by using a population of models varying different ionic conductances and extracellular ionic concentrations on a cellular model of chronic AF human atria cardiomyocyte and the anatomical variability was implemented by using models with two different tissue sizes.

This study revealed that simulated dilated atria presented more profiles maintaining reentry, therefore confirming the hypothesis that larger tissues are more prone to fibrillate, an effect that has already been shown at the clinical level (Zou et al., 2005; Qureshi et al., 2014).

### Drug Effect on the Population of Models

The population of models was not only evaluated at basal conditions but also under the effect of three different cardiovascular drugs: flecainide, verapamil, and isoproterenol. Overall pharmacological effects of the implemented drugs matched their clinical characteristics, mainly exhibiting antiarrhythmic effects in the case of flecainide and verapamil (Klein et al., 1979; Echt and Ruskin, 2020). It is interesting to point out that, in the case of dilated tissue experiments, the proportion of non-inducible AF in simulations with flecainide or verapamil was lower than for the smaller tissue size profiles. Furthermore, whereas flecainide and verapamil exhibited mainly an antiarrhythmic effect, isoproterenol exhibited a proarrhythmic effect (Oral et al., 2008). However, some antagonistic effects were observed as exhibited in Figures 3, 4, following the same behavior observed at a clinical level where undesirable effects of antiarrhythmic compounds have been identified in a minority of patients (Nogales Asensio et al., 2007). This confirms our hypothesis that the effect of the drug can be different depending on the specific expression of the ionic currents. Antagonistic effects of antiarrhythmic drugs have previously been studied (Donnelly, 2004) stating the need of identifying the different factors that can lead to this response such as genetics or drug dynamics, which are not considered in this study. However, none of these approaches analyzed the effects on a population of models of the atria.

Furthermore, we observed that the profiles that changed its behavior when adding a drug were similar in the case of the antiarrhythmic drugs simulated in this study. This suggests that specific groups with similar characteristics have analogous responses.

### Artificial Intelligence Algorithms for Arrhythmia Maintenance Prediction

As the number of simulations reached a significant number of samples with an increasing information volume, AI algorithms were applied to reveal patterns in the data. AI application in clinical environments is exponentially increasing and leading toward new diagnostic and treatment techniques (Feeny et al., 2020; Sánchez de la Nava et al., 2021). This trend has also been implemented in the electrophysiology field (Muffoletto et al., 2021; Siontis and Friedman, 2021), in which the use of algorithms has been used for detecting or evaluating proarrhythmicity (Shao et al., 2018; Halfar et al., 2021), classifying different rhythms (Wasserlauf et al., 2019) or automatizing tasks as segmentation (Yang et al., 2017). Moreover, its use in safety pharmacology could be applied to analyze all the data produced by *in silico* simulations. Particularly, the Random Forest algorithm grouped similar profiles with the same outcome, therefore implementing an AI-driven algorithm able to predict, based on the ionic combinations of each profile, the probability of AF inducibility with excellent predictive values (Sánchez de la Nava et al., 2021).

Data interpretability in the AI field has been demonstrated to be important, especially in the clinical field, where the understanding of the patterns found by algorithms is usually described as a black-box that does not allow to evaluate the biomarker identification and the decision outcome (Nicholson Price, 2018). In this case, the methodology used allowed us to analyze high amounts of data and to understand the clinical implications of the characteristics of each cluster. For example, the first variable that initiated the classification on the Random Forest algorithm was found to be the inward rectifier K^+^ channel that at the clinical level has a crucial role in controlling the frequency and stability of rotors responsible for AF (Pandit et al., 2005). Moreover, up-regulation of I_*K*1_ increased the ability to sustain faster and longer-lasting reentry, predisposing to the development of tachyarrhythmias (Noujaim et al., 2007).

Thus, the identification of this current as the first of the algorithm denotes the importance in the arrhythmia induction and maintenance mechanisms, as overexpression of this repolarization current has been associated with rotor acceleration as a consequence of a reduction in the action potential duration (Atienza et al., 2006; Pandit et al., 2011). The identification of this variable by the algorithm shows that the patterns are explainable and represent clinical scenarios that can be found in a real-life AF population.

This variable was followed by a succession of combinations resulting in several clusters with similar profiles associated with AF inducibility. Interestingly, from the overall implementation of the AI algorithm, two patterns were identified showing an increase in AF inducibility related to changes in extra and intracellular potassium levels and allowed clinical interpretability of the results that facilitate the understanding of the patterns found by the algorithm. A first cluster was found showing increased expression and concentration of potassium ions, which can be directly related to hyperkalemia. Another different pattern observed in a group with different profiles presented low values of both ionic expression and ionic concentration of potassium, which can be directly related to hypokalemic conditions (Guo et al., 2009). At the clinical level, these two conditions have been associated with the induction and triggering of different arrhythmias (Pandit et al., 2011; Skogestad and Aronsen, 2018; Rivera-Juárez et al., 2019; Rakisheva et al., 2020). More in detail, studies have shown that hypokalemia is an independent predictor of developing AF (Krijthe et al., 2013) and that specific cases of AF patients have been identified in whom hyperkalemia could induce malignant arrhythmias (Yan et al., 2018).

From the two main identified pathways that led to sustained AF reentry, the first cluster was associated with increased I_*K*1_, decreased diffusion, decreased extracellular potassium, and overexpression of sodium channels (Figure 5). The identified decreased diffusion can be directly related to lower conduction velocity, which has already been reported as a trigger for re-entrant foci and arrhythmia induction (King et al., 2013). Beyond the key role of I_*Na*_ determining excitability (Bezzina et al., 2001), the identification of this first cluster confirms the strong interaction between the molecular correlates of I_*Na*_ and I_*K*1_ as part of a common macro-molecular complex, where resting membrane potential hyperpolarization indirectly affect rotor frequency by modifying I_*Na*_ availability (Milstein et al., 2012; Ponce-Balbuena et al., 2018).

In the second cluster, both the ionic concentration of potassium and decreased potassium conductivity and extracellular concentration were combined with increased calcium dynamics (Current 1 and Current 2). These currents have a major role during the plateau phase of the action potential and its overexpression contributes to faster repolarization rates, shortening the action potential duration and increasing the risk of early afterdepolarizations which can give rise to the initiation of rotors and fibrillation (Cerrone et al., 2007; Nattel and Dobrev, 2012).

### Limitations

Results presented in this manuscript were based on a population of models of 149 subjects and further samples and variation ranges should be included to explore a wider population. Besides, simulations on the planes do not reflect all proarrhythmic areas present in 3D structures such as pulmonary veins or information including fiber orientation. The use of two different plane sizes did show that the arrhythmia inducibility was higher in bigger tissues but the simplicity of the model presenting constrained borders restricts the overall interpretability of the results, as the real atria contain highly complex structures that play important roles in the initiation and maintenance of AF. In addition, arrhythmia maintenance was considered for planes maintaining rotational activity for more than one cycle, but further analysis should be conducted to evaluate if self-termination occurred in a part of the profiles. Moreover, fibrotic tissue is a relevant condition that predisposes to AF and should be included in future studies and, if possible, the variables that conform to the AI algorithm should be transferable to the clinical practice using metrics such as conduction velocity or rotor dynamic biomarkers. In addition, machine learning algorithms will perform better and will show more robustness with a higher number of samples and different concentrations of each of the drugs. Finally, a greater number of drugs could be simulated in order to enlarge the field of application in which the algorithm can be used.

### Clinical Implications

The identification of specific groups or ionic characteristics that present proarrhythmic effects can be critical in the understanding of new tools for future pharmacological development. At the clinical level, this type of analysis can help to personalize the pharmacological treatments for each of the patients, therefore avoiding possible adverse effects. This study presents, as a result, a new trained algorithm that includes both anatomical and electrophysiological data to evaluate arrhythmia inducibility that is presented as a proof of concept for drug effect evaluation in AF, identifying similar results when compared to the clinic where dilated atria and specific cases of adverse drug effects (Bassett et al., 1997). However, this study includes the implementation of the algorithm based on parameters that are currently difficult to obtain, therefore limiting its current application at the clinical level. New biomarkers describing the ionic characteristics at the patient level should be obtained in order to apply this algorithm.

## Conclusion

Safety pharmacology has evolved including *in silico* studies that predict and classify drugs attending to the risk of causing arrhythmias. In this study, we presented a population of models approach in which arrhythmia induction was evaluated by modifications in tissue size and drug administration. A higher probability of induction was observed in larger tissue and, interestingly, antagonistic effects were observed for some of the profiles, showing that for a minority of cases, the drugs may present adverse or non-desired effects. In conclusion, we present an AI algorithm as a novel tool for pattern identification and analysis of the effect of antiarrhythmic drugs on a heterogeneous population of patients with AF.

## Data Availability Statement

The raw data supporting the conclusions of this article will be made available by the authors, without undue reservation.

## Author Contributions

FA and AS designed the study and drafted the manuscript. AS implemented the algorithms and performed the *in silico* experiments. FA, ÁA, FF-A, and AS processed, analyzed and interpreted the data, and revised the manuscript critically. All authors approved the final version of the manuscript.

## Conflict of Interest

FA and FF-A have equity from Corify Care SL. FA served on the advisory board of Medtronic and Microport. The remaining authors declare that the research was conducted in the absence of any commercial or financial relationships that could be construed as a potential conflict of interest.

## Publisher’s Note

All claims expressed in this article are solely those of the authors and do not necessarily represent those of their affiliated organizations, or those of the publisher, the editors and the reviewers. Any product that may be evaluated in this article, or claim that may be made by its manufacturer, is not guaranteed or endorsed by the publisher.
