# Supplementary material for: Artificial Intelligence-Driven Algorithm for Drug Effect Prediction on Atrial Fibrillation: An in silico Population of Models Approach
Source: Front Physiol. 2021 Dec 6;12:768468. doi: 10.3389/fphys.2021.768468 (PMC8685526; doi:10.3389/fphys.2021.768468)
Supplement: Supplementary file 1 [file Data_Sheet_1.DOCX]

Supplementary Material


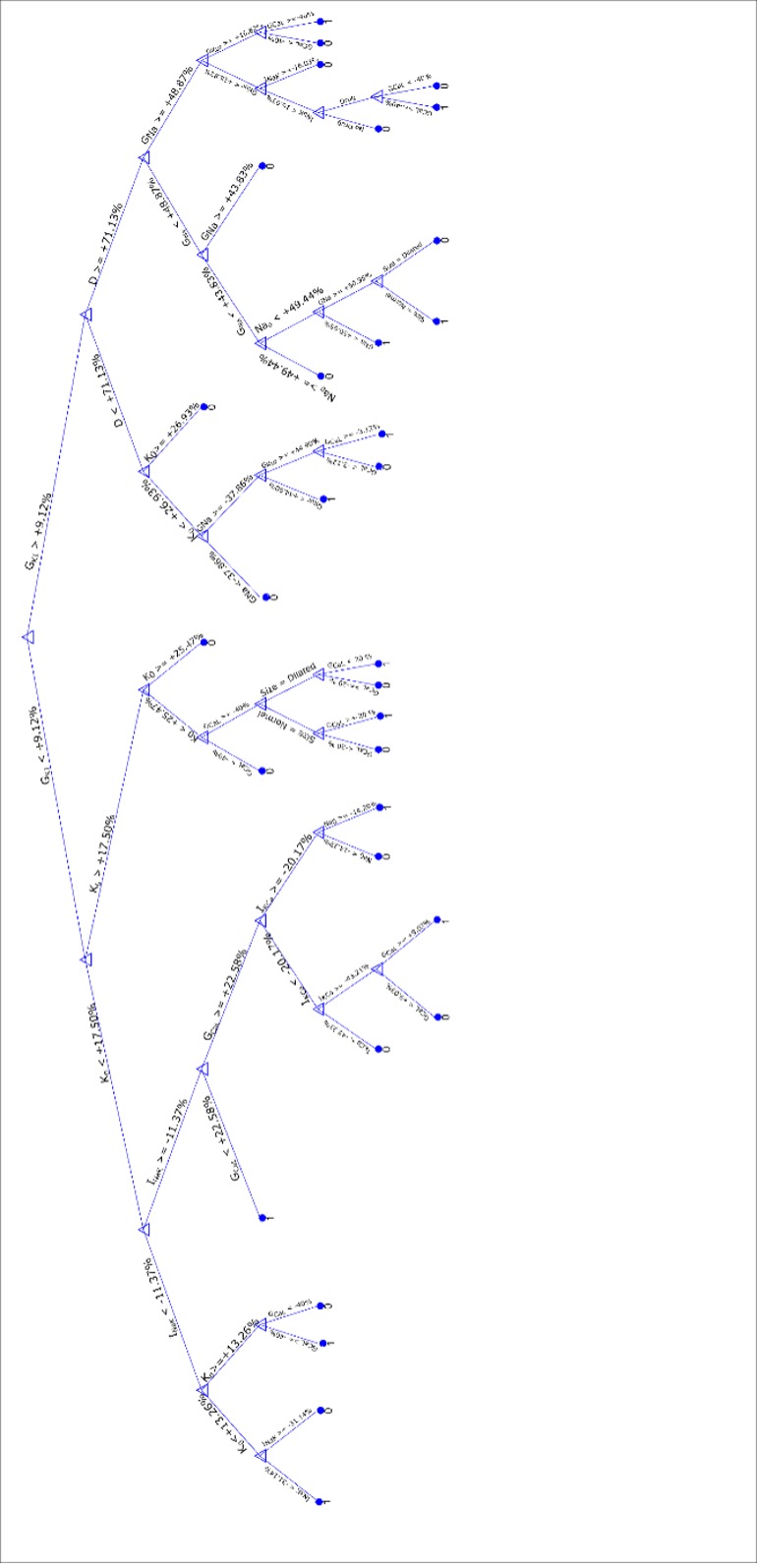


**Supplementary Figure 1:** Complete decision tree showing variables and threshold decision parameters.


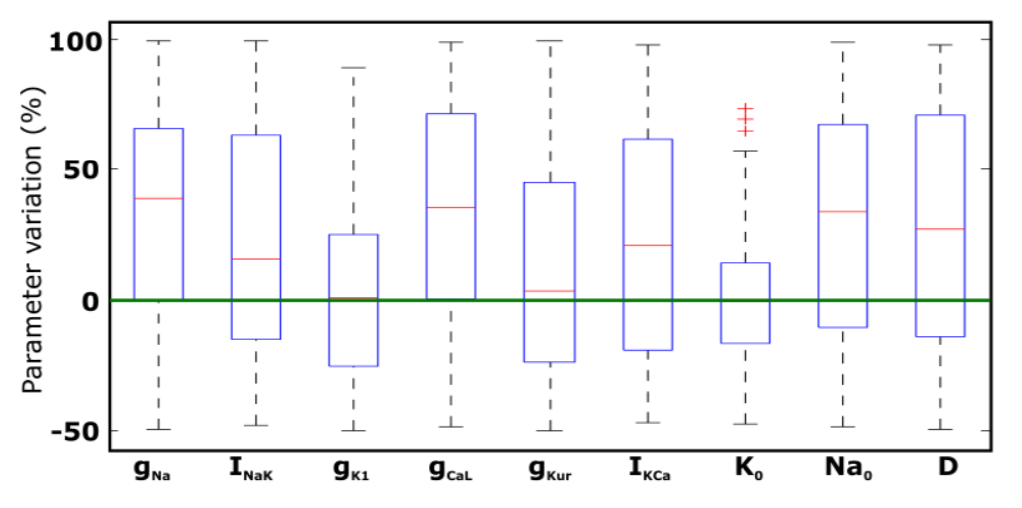


**Supplementary Figure 2:** Box plot showing the range and mean value of the varied parameters. Edges of the box represent the 1^st^ an 3^rd^ quartiles. The baseline model value is represented by the green line. A wide range of values for the diffusion coefficient, ionic conductances and concentrations is covered, being [K^+^]_0_ the most restrictive parameter.

|  | **Minimum Value** | **Maximum Value** |
| --- | --- | --- |
| **APD90 (ms)** | 140 | 330 |
| **APD50 (ms)** | 30 | 180 |
| **APD20 (ms)** | 1 | 75 |
| **APA (mV)** | 80 | 130 |
| **RMP (mV)** | -85 | -65 |
| **V20 (mV)** | -30 | 20 |

**Supplementary Table 1**: Human Atrial Action Potential biomarker ranges in AF.
